# Augmentation of peripheral lymphocyte-derived cholinergic activity in patients with acute ischemic stroke

**DOI:** 10.1186/s12883-019-1481-5

**Published:** 2019-10-15

**Authors:** Meng Yuan, Bin Han, Yiping Xia, Ye Liu, Chunyang Wang, Chao Zhang

**Affiliations:** 1 Department of Neurology, Tianjin Neurological Institute, Tianjin Medical University General Hospital, Tianjin, 300052 China; 2 Department of Neurology, The Affiliated Hospital of Qingdao University, Qingdao, 266000 China; 3 Laboratory of Physical and Chemical Research, Tianjin Centers for Disease Control and Prevention, Tianjin, 300052 China

**Keywords:** Ischemic stroke, Acetylcholine, Inflammation

## Abstract

**Background:**

Brain ischemia activates the parasympathetic cholinergic pathway in animal models of human disease. However, it remains unknown whether activation of the cholinergic pathway impacts immune defenses and disease outcomes in patients with ischemic stroke. This study investigated a possible association between peripheral cholinergic activity, post-stroke infection, and mortality.

**Methods:**

In this study, we enrolled 458 patients with acute ischemic stroke (< 24 h after onset), 320 patients with ischemic stroke on day 10, and 216 healthy subjects. Peripheral cholinergic activity, reflected by intracellular acetylcholine (ACh) content in human peripheral blood mononuclear cells (PBMCs), was determined by ultra-performance liquid chromatography-tandem mass spectrometry (UPLC-MS/MS). Expression of acetylcholinesterase (AChE) and choline acetyltransferase (ChAT) was measured by quantitative real-time PCR and western blot. Regression analyses were used to assess associations between peripheral cholinergic function and clinical outcomes.

**Results:**

Within 24 h after the onset of acute ischemic stroke, there was a rapid increase in peripheral cholinergic activity that correlated with brain infarction volume (*r* = 0.67, *P* < 0.01). Specifically, lymphocyte-derived ACh levels were significantly higher in stroke patients with pneumonia (0.21 ± 0.02 ng/10^6^ PBMC versus 0.15 ± 0.01 ng/10^6^ PBMC, *P* = 0.03). Of note, lymphocytic AChE catalytic activity was significantly lower in these patients. One-year mortality was significantly greater in patients with higher intracellular ACh levels within the first 24 h after acute stroke.

**Conclusions:**

Lymphocytes produced increased amounts of ACh in patients with acute stroke, and pneumonia was a likely result. The association between this enhanced cholinergic activity and increased risk of pneumonia/mortality suggests that increased cholinergic activity may contribute to fatal post-stroke infection.

## Background

Brain ischemia activates neurogenic pathways that lead to the inhibition of immune function by input from the sympathetic nervous system [1, 2]. Overactivation of the sympathetic nervous system and release of norepinephrine are important mediators of stroke-induced immune suppression, which predisposes patients to infection [3, 4]. In contrast, less is known about whether and how the parasympathetic nervous system may contribute to altered systemic immunity after stroke. The cholinergic pathway may affect airway hyper-reactivity, and modulation of this pathway is a possible therapeutic target in lung diseases [5, 6]. Recent preclinical evidence suggests that activation of the parasympathetic peripheral cholinergic pathway may suppress innate immunity and participate in post-stroke pneumonia in mice [7]. This potentially important link between the activation of the cholinergic pathway and immune suppression in stroke patients needs verification.

Acetylcholine (ACh) is a key neurotransmitter that mediates cholinergic input. Unlike in the CNS, in the periphery non-neuronal cells including lymphocytes are a critical source of ACh that may directly suppress immune cells in lymphoid organs. Previous studies indicated that ACh-producing lymphocytes suppress tissue macrophages and inhibit their production of pro-inflammatory cytokines [8, 9]. However, still unknown are whether ACh-producing lymphocytes may impact the systemic immune response after stroke.

In the present study, we used the previously established method of stable isotope-dilution, ultra-performance liquid chromatography-tandem mass spectrometry (UPLC-MS/MS) to focus on the relationship between intracellular ACh in lymphocytes and acute ischemic stroke [10]. Our results revealed a clear association between ACh-producing lymphocytes and the suppression of peripheral immune responses after stroke. This outcome suggests a potential contribution of ACh-producing lymphocytes to post-stroke infection and mortality in patients.

## Methods

### Subject enrollment and study design

A total of 458 patients who had experienced acute ischemic stroke onset less than 24 h before treatment were eligible for recruitment into the study. Routine cerebral computer tomography (CT) diagnostic images were acquired in all patients on a 64-row, multi-slice CT scanner (Siemens Medical Systems, München, Germany) and 163 patients also underwent 3.0 T MRI within 24 h (GE Healthcare, Marlborough, USA). All enrolled patients were over 18 years of age and gave informed consent before entering the study. In addition, enrolled patients had clinical symptom scores of ≥5 in the National Institutes of Health Stroke Scale (NIHSS). Exclusion criteria were trauma or a previous invasive procedure, cerebral hemorrhage, history of malignant tumor, chronic inflammatory disease, any infection before acute ischemic stroke, autoimmune disease, or a coagulation disorder. None of the patients had received t-PA thrombolytic treatment. The enrolled stroke patients were evaluated using the Oxfordshire Community Stroke Project (OCSP) classification criteria: lacunar infarct (LACI), total anterior circulation infarct (TACI), partial anterior circulation infarct (PACI), and posterior circulation infarct (POCI) [11]. Pneumonia was the only post-stroke infection noted, and was defined by the following two positive criteria during in-hospital stay: (1) presence of clinical and laboratory or radiological signs of pneumonia (productive cough, fever, apnea, leukopenia of < 4 × 10^9^/L, or leukocyte count of > 12 × 10^9^/L) and infiltration confirmed by chest X-ray; and (2) plasma high sensitivity C-reactive protein (hsCRP) of more than 5 mg/L. To compare peripheral cholinergic activity in acute ischemic stroke (within 24 h of onset) with that in recovery phase of stroke, lymphocyte-derived ACh was also determined on day 10 after stroke onset in the same patient. As control subjects, we recruited 216 healthy individuals who were neurologically intact and lacked any history of stroke, myocardial infarction, or peripheral artery disease. Controls approximately matched stroke patients for age and sex. This study was approved by Medical Ethical Committee and the Scientific Research Committee of Tianjin Medical University General Hospital. Written informed consent was obtained from all subjects.

### Brain MRI assessment

3 T MRI was performed with an eight-element phased-array torso coil. Lesion volumes were measured using diffusion-weighted imaging (DWI). The parameters for brain DWI at b = 1000 s/mm^2^ were as follows: TR =7059/7059 ms; TE = 45.7/64.9 ms; DFOV = 42 cm; 128 × 128 matrix directions of the motion-probing gradient, three orthogonal axes. Infarction areas were selected as regions of interest (ROIs). The images were transmitted to a workstation and FuncTool GE Software was used for image processing.

### Lymphocyte-derived ACh determination using UPLC-MS/MS

We have successfully used performance liquid chromatography-tandem mass spectrometry (UPLC-MS/MS) with Waters CORTECS chromatographic column previously to determine intracellular ACh in human PBMCs with detection limits of up to 0.005 ng/10^6^ PBMCs [10]. In brief, in this study fresh PBMCs were prepared from blood and added to 100 μL deionized H_2_O/0.1% formic acid (vol:vol), and then vortexed to produce homogenates. The homogenates were further broken down ultrasonically and were de-proteinized by adding 300 μL acetonitrile containing the internal standard (D9-ACh, C/D/N, Quebec, Canada). The sample was vortexed again for 1 min before being centrifuged at 15,000 rpm for 10 min [12]. The supernatant was then filtered and transferred to an autosampler glass vial for ACh determination. UPLC–MS/MS analyses were carried out using an Acquity UPLC system coupled to a Xevo TQ-S (Waters Corporation, Milford, MA, USA) consisting of a binary pump, an automatic sampler, and a column compartment. An Acquity UPLC CORTECS HILIC column (1.6 μm, 100 mm × 2.1 mm) was used to ensure sufficient efficacy of isolation and ionization of ACh. The column temperature was set to 45 °C and the flow rate was 0.4 mL/min. The solvents were 50 mmol/L ammonium formate (solvent A, pH adjusted to 3 with formic acid) and acetonitrile (solvent B) with a gradient over the 3.5 min run time as follows: 20% A (initial), 20–50% A (0–0.75 min), 50–70% A (0.75–1.25 min), 70–70% A (1.25–1.7 min), 70–20% A (1.7–2.1 min), 20–20% A (2.1–3.5 min). The sample injection volume was 5 μL. ACh and D9-ACh were identified by the triple quadrupole mass spectrometer using positive electrospray ionization (ESI) mode. Assays were validated according to the FDA Bioanalytical Method Validation Guidance for Industry [13].

### Assays of lymphocytic vesicular ACh transporter (VAChT), acetylcholinesterase (AChE), and choline acetyltransferase (ChAT)

PBMC total mRNA was extracted using the RNeasy Mini Kit (Qiagen, Hilden, Germany). Reverse transcription reactions were prepared using the SYBR Premix Ex Taq System (Takara, Japan). Real-time PCR was performed with the IQ5 System (Bio-Rad, US). Cycle conditions and relative quantification were carried out following the manufacturer’s instructions. Expression of VAChT, AChE, and ChAT were calculated using the comparative computerized tomography method with efficiency calculations, and with all mRNA levels normalized to endogenous GAPDH mRNA. Amplification of the targets was performed using the following primers: 5′- GGCATAGCCCTAGTCGACAC − 3′ (forward) and 5′- CGTAGGCCACCGAATAGGAG − 3′ (reverse) for VAChT, 5′- GGGTGGTAGACGCTACAACC − 3′ (forward) and 5′- GTGCCCTCAAAACCTGGGTAT − 3′ (reverse) for AChE, 5′- AACCACGGAGATGTTCTGCTGCTAT − 3′ (forward) and 5′- TTGTTGCCAATGGCTTGCTCTCAG − 3′ (reverse) for ChAT. All reactions were run in triplicate. Protein was obtained using protein extraction reagent containing protease inhibitors. SDS protein electrophoresis and western blotting were performed following standard protocols. The antibodies used were rabbit anti-AChE (1:1000, Abcam, Cambridge, USA), goat anti-ChAT (1:500, Merck Millipore (Chemicon), Darmstadt, Germany), and goat anti-VAChT (1:1000, Abcam). Blots were visualized using the SYNGENE imaging system (UK) and analyzed with Image J software. AChE catalytic activity was measured with reference to Ofek’s method [14]. To eliminate any assay-by-assay variation, we reanalyzed 10 arbitrary control samples from each cohort in different plates and at different measurement times. The capacity of hydrolyzing substrate was represented by OD value per 100 nmol substrate/min × mL.

### Statistical analysis

Statistical analysis was performed using SPSS Statistics 20 (IBM Corp. Released 2011; IBM SPSS Statistics for Windows, Version 20.0, Armonk, NY, USA). Subject characteristics are presented as means ± SD; all other results are expressed as means ± SEM. To test for significant differences between the groups, a one-way ANOVA was applied. Post hoc testing was performed using the Bonferroni correction. From individual subject data, correlation coefficients were calculated to test for associations between selected parameters (i.e., ACh concentration, age, sex percentage). We used a logistic regression model to estimate the impact of clinical factors on OCSP stroke subtypes. The Kaplan–Meier estimator was used to determine the outcome risk at 1 year after the onset of acute ischemic stroke. In all tests, the threshold for significance was set at *P* < 0.05.

## Results

### Subject characteristics

The baseline characteristics of the subject population are shown in Table 1, along with results from routine blood and neurological tests. Stroke patients and control subjects were not significantly different in demographic traits, medical history, or plasma lipid values. Of all 458 patients, 4 patients died in 10 days and 134 patients failed to draw blood because of early discharge from the hospital. Thus re-determination of PBMC-derived ACh was performed in 320 patients in the recovery phase of stroke (10 days after stroke). 210 of 320 patients had been followed-up till 30 days after acute stroke. So we could gain dynamics of peripheral cholinergic activity from onset of acute stroke to 30 days after stroke. 94 patients (20.5% of the total) were diagnosed with pneumonia within 24 h after acute stroke. In the acute ischemic stroke (< 24 h) group, the mean hsCRP was 10.12 mg/L, and this was significantly higher than that in controls and in patients 10 days after stroke (*P* = 0.029). Those with acute ischemic stroke also had significantly elevated white blood cell counts compared with the other two groups (*P* = 0.033). Median baseline NIHSS and modified Rankin Scale (mRS) were 8 (interquartile range, IQR, 2–24) and 3 (IQR, 1–5), respectively, in the acute stroke group. These scores were significantly decreased on day 10 on after acute stroke (*P* < 0.001) (Table 1).

**Table 1:** Subject Characteristics

	Control (*n* = 216)	Stroke within 24 h (*n* = 458)	Stroke on day 10 (*n* = 320)	*p*
Demographic				
Age, y, mean (SD)	68.14 (0.89)	67.56 (1.65)	67.94 (1.15)	0.596
Male gender, n (%)	121 (56)	243 (55)	208 (65)	0.258
Medical history, n (%)				
Hypertension	63 (29.2)	142 (31.0)	104 (32.5)	0.705
DM	69 (31.9)	103 (22.5)	90 (28.8)	0.626
History of smoking	52 (24.1)	90 (19.7)	91 (28.4)	0.229
History of alcohol	43 (20.0)	110 (24.0)	70 (21.9)	0.926
Plasma lipids				
TC, mmol/L (SD)	4.68 (0.76)	4.70 (0.84)	4.50 (0.51)	0.586
HDL, mmol/L (SD)	1.19 (0.08)	1.12 (0.09)	1.32 (0.02)	0.732
LDL, mmol/L (SD)	2.89 (0.05)	2.95 (0.54)	2.91 (0.24)	0.510
TG, mmol/L (SD)	1.53 (0.56)	1.41 (0.62)	1.29 (0.21)	0.675
Inflammatory biomarkers				
hsCRP, mg/L (SD)	1.98 (0.35)	10.12 (0.58)	3.56 (0.51)	0.029*
WBCC, 10^3^/ml (SD)	5.4 (0.8)	9.8 (0.8)	4.8 (0.7)	0.033*
D-dimer, ng/mL (SD)	549.84 (85.84)	697.92 (181.48)	642.84 (92.81)	0.184
Fibrinogen, g/L (SD)	2.49 (0.17)	2.72 (0.11)	2.92 (0.03)	0.849
Stroke subtype, n (%)				0.449
Large artery atherosclerosis		188 (41.0)	124 (38.7)	
Microangiopathy		90 (19.7)	56 (17.5)	
Cardioembolism		126 (27.5)	102 (31.9)	
Unknown		54 (11.8)	38 (11.9)	
Stroke syndrome				0.346
LACS		126 (27.5)	43 (13.4)	
TACS		53 (11.6)	57 (17.6)	
PACS		174 (38.0)	131 (41.2)	
POCS		105 (22.9)	89 (27.8)	
Neurological scores at admission, median (IQR)				
NIHSS		8 (2–24)	2 (0–6)	< 0.001*
mRS		3 (1–5)	1 (0–2)	< 0.001*

*DM* diabetes mellitus, *TC* total cholesterol, *HDL* high-density lipoprotein, *LDL* low-density lipoprotein, *TG* total triglyceride, *hsCRP* high-sensitivity C-reactive protein, *WBCC* white blood cell counts, *ESR* erythrocyte sedimentation rate, *LACS* lacunar stroke syndrome, *TACS* total anterior circulation stroke syndrome, *PACS* partial anterior circulation stroke syndrome, *POCS* posterior circulation stroke syndrome, *NIHSS* National Institutes of Health Stroke Scale, *mRS* modified Rankin Scale

### PBMC-derived ACh was elevated and correlated with brain infarct volume in stroke patients

**Fig. 1:**
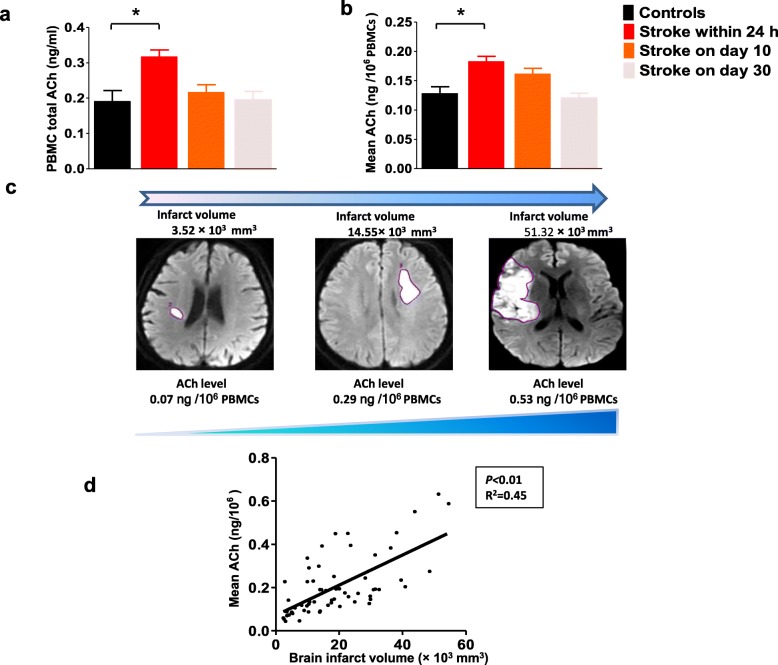
Elevated peripheral cholinergic activity associated with stroke severity and post-stroke inflammation. **a**, **b** PBMCs-derived ACh levels were significantly increased in stroke patients within 24 h after stroke, but tended to recover by 10 days and 30 days post-stroke. **c** Peripheral ACh changes in PBMCs were accompanied by increasing brain infarction volume. **d** ACh levels were significantly correlated with brain infarction volume

Using UPLC-MS/MS, we confirmed the stable presence of ACh in PBMCs and quantitatively determined intracellular ACh content. Overall, total ACh content in PBMCs tended to be higher in patients with ischemic stroke compared with control subjects. However, *post-hoc* analysis revealed that, compared with controls, ACh concentrations were only statistically different within 24 h of stroke onset (*P* = 0.016) (control: 0.19 ± 0.03 ng/mL, within 24 h of stroke: 0.32 ± 0.02 ng/mL; 10 days after stroke: 0.20 ± 0.02 ng/mL; 30 days after stroke: 0.18 ± 0.02 ng/mL; *P* < 0.01; Fig. 1a). Furthermore, results were similar when comparing ACh concentrations per 10^6^ PBMCs (control: 0.11 ± 0.01 ng/10^6^ cells; within 24 h of stroke: 0.18 ± 0.01 ng/10^6^ cells; 10 days after stroke: 0.15 ± 0.01 ng/10^6^ cells; 30 days after stroke: 0.11 ± 0.01 ng/10^6^ cells; *P* = 0.011; controls versus within 24 h of stroke: *P* = 0.002; Fig. 1b)**.** Importantly, we also found that, with an increase in brain infarction volume, PBMC-derived ACh levels increased dramatically (Fig. 1c). When we analyzed individual patients, ACh concentrations per 10^6^ PBMCs correlated significantly with brain infarction volume (Fig. 1d, Pearson *r* = 0.67, R^2^ = 0.45, *P* < 0.01).

### Cholinergic changes after acute ischemic stroke, especially in patients with pneumonia

**Fig. 2:**
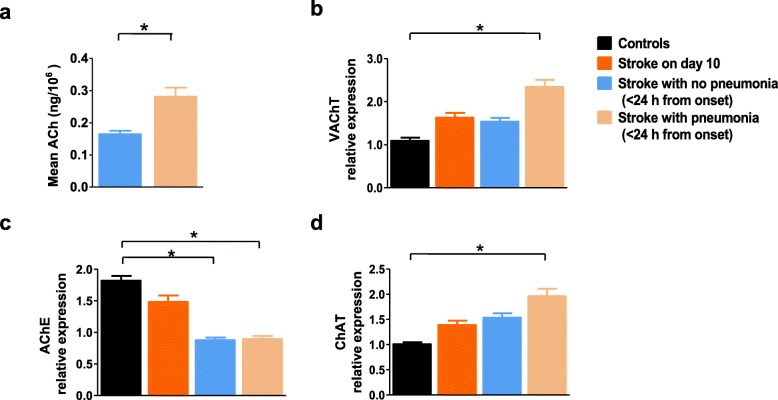
Altered cholinergic activity after acute ischemic stroke, especially in patients with pneumonia. **a** Within 24 h of stroke, patients with stroke-induced pneumonia had higher ACh concentrations than their counterparts without pneumonia. **b**–**d** Intracellular VAChT, AChE, and ChAT relative protein expression of PBMCs in stroke patients. VAChT and ChAT protein was only upregulated in PBMCs in acute ischemic stroke patients with pneumonia. Intracellular AChE activity showed a trend toward a decrease in acute ischemic stroke. Levels of protein expression by western blot are relative to the amount of GAPDH

Blood collection was done before pneumonia occurred in all of 94 patients with pneumonia within 24 h of stroke. During the follow-ups, 52 patients had documented episodes of emesis, of whom 41 had bulbar palsy and 11 impaired consciousness. These patients with a clear mechanism that could explain their pneumonia were excluded in the analysis of the association of peripheral cholinergic activity with immuno-compromised pneumonia. Within 24 h of stroke onset, patients with stroke-induced pneumonia in 42 patients had higher ACh concentrations than their counterparts without pneumonia (Fig. 2a, 0.28 ± 0.03 ng/10^6^ cells versus 0.16 ± 0.01 ng/10^6^ cells; *P <* 0.01). The relative expressions of both VAChT and AChE mRNA decreased significantly in patients with acute stroke, regardless of concomitant pneumonia (*P* < 0.01; Additional file 1 a–b). Represented as OD values, AChE activity was essentially similar in controls (0.034 ± 0.008 OD) and patients on day 10 after stroke onset (0.017 ± 0.003 OD), but was significantly lower in patients with acute ischemic stroke both with and without pneumonia (0.014 ± 0.005 OD and 0.012 ± 0.004 OD; *P* < 0.01; Additional file 1c). However, PBMCs in patients with acute ischemic stroke underwent modulation of ChAT mRNA expression, resulting in levels indistinguishable from those of subjects grouped as controls or patients on post-stroke day 10 (*P* = 0.73; Additional file 1d). Western blot of these proteins showed similar changes in AChE but not VAChT (*P* < 0.01; Fig. 2b, c). However, ChAT protein expression was upregulated in PBMCs in patients with acute ischemic stroke and pneumonia, but not in controls, patients on day 10 post-stroke, or patients with acute stroke but not pneumonia (*P* = 0.04 and *P* = 0.03, respectively; Fig. 2d). These results support the idea that inflammation, such as that which occurs in pneumonia, is accompanied by activated peripheral lymphatic cholinergic tone.

**Fig. 3:**
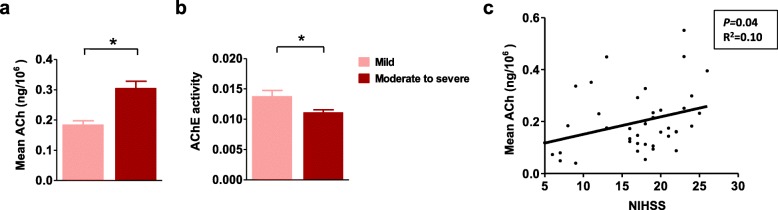
Elevated cholinergic activity was associated with disability in acute stroke patients with pneumonia. The patients were stratified into two subgroups, mild stroke (NIHSS≤15) and moderate/severe stroke (NIHSS> 15). **a** Higher cholinergic activity was detected in patients with moderate /severe stroke than mild stroke. **b** Moderate/severe stroke patients had decreased lymphocytic AChE activity. **c** Peripheral lymphocyte-derived ACh was positively correlated with the severity of stroke with pneumonia

To further explore the relationship between peripheral cholinergic activity and stroke severity, we divided the patients with pneumonia into two subgroups: 11 patients with mild stroke (NIHSS≤15) and 31 patients with moderate/severe stroke (NIHSS> 15). We found that patients with moderate /severe stroke had significantly higher cholinergic activity than mild stroke (Fig.3a, 0.18 ± 0.01 ng/10^6^ cells versus 0.29 ± 0.03 ng/10^6^ cells; *P <* 0.01), accompanied by decreased AChE activity (Fig.3b, 0.013 ± 0.005 OD and 0.011 ± 0.003 OD; *P* = 0.026) and AChE expression (Additional file 2a). But there were no differences in VAChT and ChAT expression between the two subgroups of patients (Additional file 2b and c). Strikingly, peripheral lymphocyte-derived ACh was positively correlated with the severity of stroke (Fig. 3c, Pearson *r* = 0.32, R^2^ = 0.10, *P* = 0.04). Patients who suffered from more severe disability had higher levels of peripheral cholinergic activity.

### Association of lymphocyte-derived ACh with clinical subtype and 1-year mortality

**Fig. 4:**
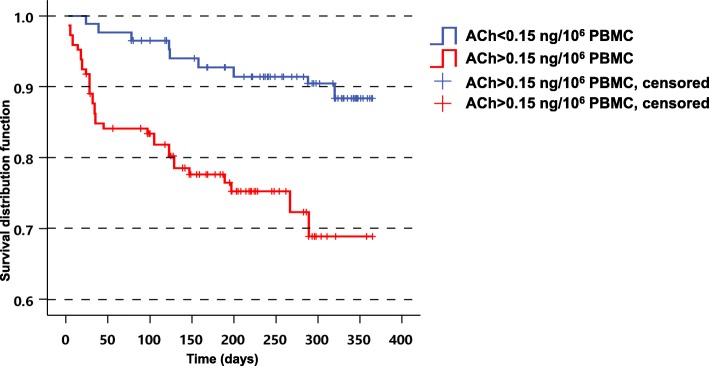
Kaplan–Meier estimates of 1-year survival stratified by ACh level in acute ischemic stroke patients

To test our primary hypothesis that lymphocyte-derived ACh might be related to OCSP subtype, we performed correlative analyses. Patients were divided into two groups: anterior and posterior circulation infarction. Multivariate linear regression analyses revealed a statistically significant association in lymphocyte-derived ACh between patients monitored within 24 h of stroke onset and posterior circulation infarction (*P* = 0.027; Table 2) but not anterior circulation infarction (Additional file 3). Additionally, we dichotomized initial ACh levels within 24 h of stroke at the upper 2nd and 3rd tertiles of ACh combined, corresponding to an ACh level ≥ 0.15 ng/10^6^ cells. Interaction terms were added to the models to test the modifying effects of confounding variables on the association between ACh level and 1-year all-cause mortality using the likelihood-ratio test. At 1-year follow-up, 62 subjects (13.5%) had died. The mean ACh level at initial testing was higher in patients who had died (0.38 ± 0.02 vs. 0.24 ± 0.02 ng/10^6^ cells, *P* < 0.001), and 1-year survival was higher in patients with low ACh levels (Fig. 4). After adjustment for age, sex, and NIHSS score, high ACh levels (dichotomized at the upper 2nd and 3rd tertile of their distribution) were significantly associated with 1-year mortality (HR = 1.75; 95% CI:1.08–3.14; *P* < 0.001). This indicated that initial higher ACh levels in acute ischemic stroke (within 24 h) may be an independent predictor of mortality at 1 year.

**Table 2:** Multivariate Logistic Regression Analysis with Backward Elimination of Factors Independently Associated With Posterior Circulation Infarction

Independent Variable	OR (95% CI)	*P* value
ACh	0.210 (0.122–0.568)	0.027
CRP	11.232 (0.982–57.232)	0.307
Pneumonia	0.027 (0.005–0.058)	0.404
NIHSS	8.324 (2.546–32.152)	0.128
FIB	0.106 (0.045–0.168)	0.038

*ACh* acetylcholine, *CRP* C-reactive protein, *NIHSS* National Institutes of Health Stroke Scale, *FIB* fibrinogen

## Discussion

Results from the present study document stroke-induced activation of the parasympathetic cholinergic anti-inflammatory pathway, which may be related to post-stroke mortality. These two pathways, the nervous and immune systems, are closely interconnected via intense bidirectional communication [15]. The parasympathetic vagus nerve and the neurotransmitter ACh have been previously shown to act as a negative feedback loop to prevent potentially harmful pro-inflammatory responses. Vagal stimulation can mediate anti-inflammatory properties by stimulating splenic T cells to produce ACh, which binds to α7 nicotinic acetylcholine receptors (α7nAChR), thus downregulating TNF-α produced by activated macrophages [8]. The spleen links efferent vagus nerve signals and peripheral non-neural cholinergic function in immune cells. With the inflammatory cholinergic reflex, ACh-producing lymphocytes might then be released from the spleen into the blood. If this is the case, there is little doubt that lymphocyte-derived ACh is a direct marker of peripheral immune cholinergic activity. Previous studies have indicated that lymphocytic cholinergic activity is altered in animal models of immunological abnormalities [16]. We therefore postulated that ACh levels are the result of equilibrium between ACh released directly or indirectly by the vagus nerve through its effects on ACh-producing lymphocytes and ACh degradation by cholinesterase.

Here, we demonstrated elevated PBMC-derived ACh within the first 24 h in acute ischemic stroke. In line with this result, reduced AChE expression and catalytic activity were observed, suggesting an overabundance of stroke-induced cholinergic activity. Accordingly, AChE has been proposed as a marker of inflammation and a prognostic factor for recovery [17, 18]. In addition, peripheral ACh was positively associated with brain infarction volume early in the progression of stroke.

We further revealed that stroke-induced pneumonia was accompanied by a significant increase in PBMC-derived ACh. This result highlights the role of cholinergic modulations as early facilitators in the peripheral response to a pulmonary infection. Immunosuppression may be induced not only by overactivated cholinergic functions, but also by activation of the sympathetic nervous system that stimulates splenic immune cells to release ACh. Early in the relay, ACh amplifies vagal cholinergic signaling at the macrophage level and suppresses immune responses in the lung [4, 18]. The coexistence of cholinergic and noradrenergic receptors on lymphocytes reflects an interaction between the sympathetic and parasympathetic systems. In the present study, we found that increased parasympathetic activity after acute stroke mediated susceptibility to bacterial pneumonia. Interestingly, ACh level within 24 h of stroke onset was an important predictor of prognosis in our study. The association between initial ACh levels and NIHSS may provide an additional insight into post-stroke outcomes. Thus, outcome after stroke may be influenced by hyperactivity of the HPA axis, feedback of the cholinergic pathway, or destruction of discrete brain regions. A patient’s ACh level may therefore be used as an early predictor of stroke-associated survival time, at least to some extent. Although stimulation of the cholinergic pathway via the vagus nerves may be a neuroprotective strategy [19–21], such stimuli might also exacerbate the risk of life-threatening medical complications after stroke [4].

Some limitations to our study should be considered. First, the patient sample was relatively small, although it was carefully selected. Therefore, the results of this study need confirmation in a larger cohort. Second, stroke patients in this study did not undergo any t-PA treatment and our outcomes are therefore not likely to be fully comparable to those of populations that received thrombolytic treatment.

## Conclusions

This study demonstrates that the spleen undergoes profound changes during acute ischemic stroke, a finding that provides direct evidence for activation of the cholinergic anti-inflammatory pathway via the vagal nerve–spleen axis. Therefore, peripheral ACh may act as a prognostic marker of patients’ long-term outcome after stroke.

## Supplementary information

**Additional file 1.** Intracellular VAChT, AChE and ChAT activity in PBMCs in acute ischemic stroke. a-b The relative expression of both VAChT and AChE mRNA decreased significantly in sufferers of acute stroke within 24 h, regardless of concomitant pneumonia. c Represented as OD values, AChE activity was essentially similar in controls and patients on day 10 after stroke onset, but was significantly lower in patients with acute ischemic stroke with and without pneumonia. d ChAT mRNA expression resulting in levels indistinguishable among the groups. Levels of mRNA expression were relative to the amount of GAPDH.

**Additional file 2.** Differential expression of lymphocyte-derived cholinergic components in patients with mild stroke and moderate/severe stroke. a The relative expression of AChE mRNA decreased significantly in patients of moderate/severe stroke within 24 h, regardless of concomitant pneumonia. b-c Both ChAT and VAChT mRNA expression resulted in levels without significance between the two groups. Levels of mRNA expression were relative to the amount of GAPDH.

**Additional file 3.** Multivariate Logistic Regression Analysis with Backward Elimination of Factors that may be Associated with Anterior Circulation Infarction.

## Data Availability

The datasets analyzed during the current study are available from the corresponding author on reasonable request.

## Abbreviations

ACh: Acetylcholine
AChE: Acetylcholinesterase
AChR: Acetylcholine receptor
ChAT: Choline acetyltransferase
ChT1: High-affinity choline transporter
DWI: Diffusion-weighted imaging
PBMCs: Peripheral blood mononuclear cells
UPLC-MS/MS: Ultra-performance liquid chromatography-tandem mass spectrometry
VAChT: Vesicular acetylcholine transporter
