# Supplementary material for: Augmentation of peripheral lymphocyte-derived cholinergic activity in patients with acute ischemic stroke
Source: BMC Neurol. 2019 Oct 15;19:236. doi: 10.1186/s12883-019-1481-5 (PMC6792255; doi:10.1186/s12883-019-1481-5)

Additional file 1 **Intracellular VAChT, AChE and ChAT activity in PBMCs in acute ischemic stroke.**

**a-b** The relative expression of both VAChT and AChE mRNA decreased significantly in sufferers of acute stroke within 24 h, regardless of concomitant pneumonia. **c** Represented as OD values, AChE activity was essentially similar in controls and patients on day 10 after stroke onset, but was significantly lower in patients with acute ischemic stroke with and without pneumonia. **d** ChAT mRNA expression resulting in levels indistinguishable among the groups. Levels of mRNA expression were relative to the amount of GAPDH.


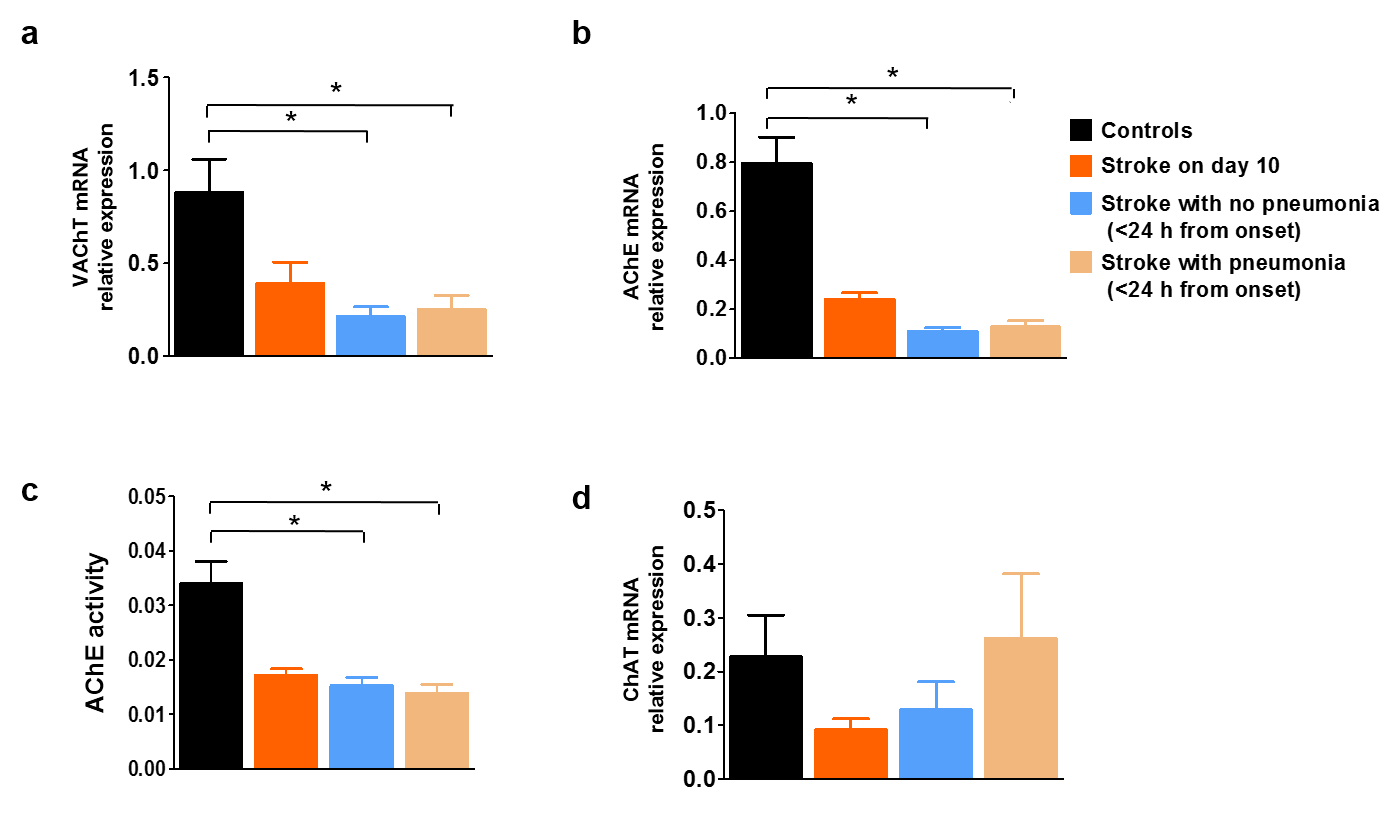

Supplement: Supplementary file 1 — Additional file 1. Intracellular VAChT, AChE and ChAT activity in PBMCs in acute ischemic stroke. a-b The relative expression of both VAChT and AChE mRNA decreased significantly in sufferers of acute stroke within 24 h, regardless of concomitant pneumonia. c Represented as OD values, AChE activity was essentially similar in controls and patients on day 10 after stroke onset, but was significantly lower in patients with acute ischemic stroke with and without pneumonia. d ChAT mRNA expression resulting in levels indistinguishable among the groups. Levels of mRNA expression were relative to the amount of GAPDH. [file 12883_2019_1481_MOESM1_ESM.docx]
