# Supplementary material for: Augmentation of peripheral lymphocyte-derived cholinergic activity in patients with acute ischemic stroke
Source: BMC Neurol. 2019 Oct 15;19:236. doi: 10.1186/s12883-019-1481-5 (PMC6792255; doi:10.1186/s12883-019-1481-5)

Additional file 2 **Differential expression of lymphocyte-derived cholinergic components in patients with mild stroke and moderate/severe stroke. a** The relative expression of AChE mRNA decreased significantly in patients of moderate/severe stroke within 24 h, regardless of concomitant pneumonia. **b-c** Both ChAT and VAChT mRNA expression resulted in levels without significance between the two groups. Levels of mRNA expression were relative to the amount of GAPDH.


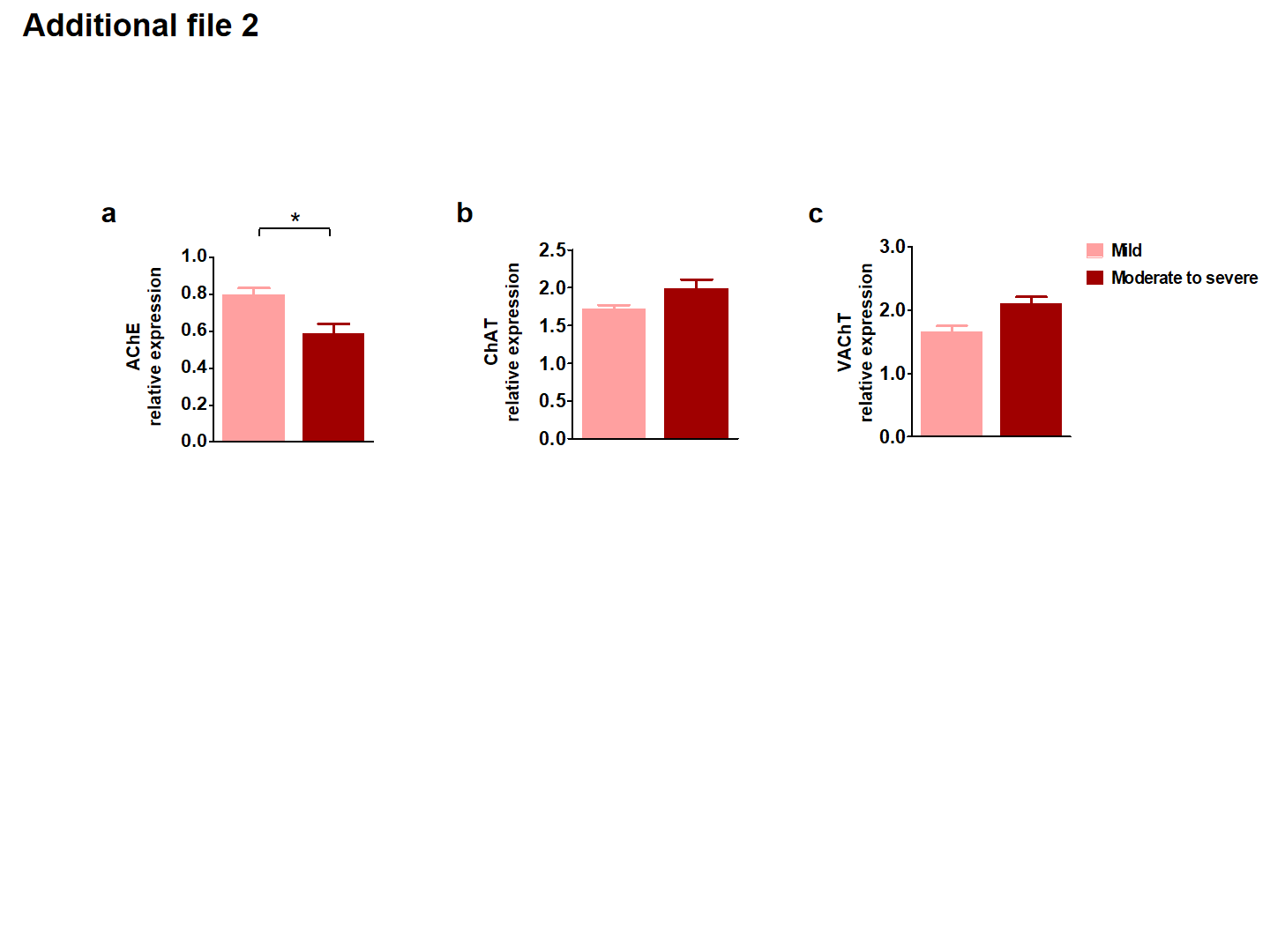

Supplement: Supplementary file 2 — Additional file 2. Differential expression of lymphocyte-derived cholinergic components in patients with mild stroke and moderate/severe stroke. a The relative expression of AChE mRNA decreased significantly in patients of moderate/severe stroke within 24 h, regardless of concomitant pneumonia. b-c Both ChAT and VAChT mRNA expression resulted in levels without significance between the two groups. Levels of mRNA expression were relative to the amount of GAPDH. [file 12883_2019_1481_MOESM2_ESM.docx]
