# Supplementary material for: Augmentation of peripheral lymphocyte-derived cholinergic activity in patients with acute ischemic stroke
Source: BMC Neurol. 2019 Oct 15;19:236. doi: 10.1186/s12883-019-1481-5 (PMC6792255; doi:10.1186/s12883-019-1481-5)
Supplement: Supplementary file 3 — Additional file 3. Multivariate Logistic Regression Analysis with Backward Elimination of Factors that may be Associated with Anterior Circulation Infarction. [file 12883_2019_1481_MOESM3_ESM.docx]

Additional file 3 **Multivariate Logistic Regression Analysis with Backward Elimination of Factors that may be Associated with Anterior Circulation Infarction**

| **Independent Variable** | | **OR (95% CI)** | ***P* value** |
| --- | --- | --- | --- |
|  | **ACh** | 0.230 (0.11-0.32) | 0.132 |
|  | **CRP** | 8.178 (1.092-23.948) | 0.461 |
|  | **Pneumonia** | 0.035 (0.003-0.042) | 0.185 |
|  | **NIHSS** | 5.482 (1.488-30.326) | 0.151 |
|  | **FIB** | 0.218 (0.145-0.468) | 0.237 |

ACh, acetylcholine; CRP, C-reactive protein; NIHSS, National Institutes of Health Stroke Scale;

FIB, fibrinogen.
